# Prediction of Intrauterine Growth Restriction and Preeclampsia Using Machine Learning-Based Algorithms: A Prospective Study

**DOI:** 10.3390/diagnostics14040453

**Published:** 2024-02-19

**Authors:** Ingrid-Andrada Vasilache, Ioana-Sadyie Scripcariu, Bogdan Doroftei, Robert Leonard Bernad, Alexandru Cărăuleanu, Demetra Socolov, Alina-Sînziana Melinte-Popescu, Petronela Vicoveanu, Valeriu Harabor, Elena Mihalceanu, Marian Melinte-Popescu, Anamaria Harabor, Elena Bernad, Dragos Nemescu

**Affiliations:** 1 Department of Mother and Child Care, “Grigore T. Popa” University of Medicine and Pharmacy, 700115 Iasi, Romania; tanasaingrid@yahoo.com (I.-A.V.); acarauleanu@yahoo.com (A.C.); demetrasocolov@gmail.com (D.S.); petronela.pintilie@yahoo.com (P.V.); emih2001@yahoo.com (E.M.);; 2 Faculty of Computer Science, Politechnica University of Timisoara, 300006 Timisoara, Romania; robert.bernad@student.upt.ro; 3 Department of Mother and Newborn Care, Faculty of Medicine and Biological Sciences, ‘Ștefan cel Mare’ University, 720229 Suceava, Romania; alina.melinte@usm.ro (A.-S.M.-P.); valeriuharabor@yahoo.com (V.H.);; 4 Clinical and Surgical Department, Faculty of Medicine and Pharmacy, ‘Dunarea de Jos’ University, 800216 Galati, Romania; melinte.marian@usm.ro; 5 Department of Internal Medicine, Faculty of Medicine and Biological Sciences, ‘Ștefan cel Mare’ University, 720229 Suceava, Romania; 6 Department of Obstetrics-Gynecology II, Faculty of Medicine, “Victor Babes” University of Medicine and Pharmacy, 300041 Timisoara, Romania

**Keywords:** preeclampsia, intrauterine growth restriction, prediction, machine learning, screening

## Abstract

(1) Background: Prenatal care providers face a continuous challenge in screening for intrauterine growth restriction (IUGR) and preeclampsia (PE). In this study, we aimed to assess and compare the predictive accuracy of four machine learning algorithms in predicting the occurrence of PE, IUGR, and their associations in a group of singleton pregnancies; (2) Methods: This observational prospective study included 210 singleton pregnancies that underwent first trimester screenings at our institution. We computed the predictive performance of four machine learning-based methods, namely decision tree (DT), naïve Bayes (NB), support vector machine (SVM), and random forest (RF), by incorporating clinical and paraclinical data; (3) Results: The RF algorithm showed superior performance for the prediction of PE (accuracy: 96.3%), IUGR (accuracy: 95.9%), and its subtypes (early onset IUGR, accuracy: 96.2%, and late-onset IUGR, accuracy: 95.2%), as well as their association (accuracy: 95.1%). Both SVM and NB similarly predicted IUGR (accuracy: 95.3%), while SVM outperformed NB (accuracy: 95.8 vs. 94.7%) in predicting PE; (4) Conclusions: The integration of machine learning-based algorithms in the first-trimester screening of PE and IUGR could improve the overall detection rate of these disorders, but this hypothesis should be confirmed in larger cohorts of pregnant patients from various geographical areas.

## 1. Introduction

Intrauterine growth restriction (IUGR) and preeclampsia (PE) are two obstetrical complications that occur mainly in the context of ischemic placental disease. PE impacts approximately 2% to 8% of pregnancies and exerts a significant toll, contributing to more than 70,000 maternal deaths and approximately 500,000 fetal demises annually [1]. Typical clinical manifestations of PE are represented by de novo hypertension after 20 weeks of gestation, proteinuria, and/or specific organ dysfunction (liver dysfunction, acute kidney injury, pulmonary edema, focal neurological manifestations, hemolysis, thrombocytopenia, etc.) [2].

PE can determine maternal complications such as HELLP (hemolysis, elevated liver enzymes, and low platelet count) syndrome or eclampsia (maternal convulsive seizures), and fetal complications such as IUGR and preterm birth. Its screening and early detection are important elements of obstetrical management that allow clinicians to offer preventive measures (administration of aspirin before 16 weeks of gestation) or to perform an individualized monitoring program [3,4,5].

IUGR can be described as the inability of the fetus to attain its inherent genetic growth potential [6]. The most important tool for IUGR screening and diagnosis is ultrasound, and the diagnostic criteria include an estimated fetal weight (EFW), <3rd percentile or EFW < 10th percentile, in combination with abnormal fetoplacental Doppler parameters [7]. IUGR itself can be accompanied by important fetal and neonatal morbidity and mortality, and the long-term consequences include a higher risk of neuropsychomotor disorders or metabolic syndrome [8,9,10].

Most international societies of obstetrics and gynecology (American College of Obstetricians and Gynecologists (ACOG), the National Institute for Health and Care Excellence (NICE), and the International Society for the Study of Hypertension in Pregnancy (ISSHP)) recommend a targeted screening of PE in the first trimester of pregnancy, in the presence of maternal risk factors, while the screening of IUGR is recommended to be universally performed, even in the absence of maternal risk factors [1,11,12,13].

Nowadays, a combined first-trimester screening of these disorders is preferred, and it includes maternal characteristics, ultrasound markers, and maternal serum biomarkers. Numerous studies have demonstrated the superiority in terms of the predictive performance of a combined screening in comparison with a screening based only on maternal risk factors, and a plethora of maternal biomarkers have been evaluated to obtain the best prediction [14,15].

PlGF (placental growth factor) is a proangiogenic marker, abundantly expressed in the placenta, vascular endothelial cells, fibroblasts, osteoblasts, smooth muscle cells, and monocytes [16]. It has been shown that low levels of PlGF are associated with the development of preeclampsia and IUGR [17,18,19].

PP-13 (placental protein-13) is another serum biomarker, a member of the galectin family involved in spiral artery remodeling and placental inflammation, which has demonstrated good predictive performance for IUGR and preeclampsia [20,21,22].

Lately, several studies indicated that predictive models based on artificial intelligence, such as artificial neural networks and machine learning-based algorithms, could improve the screening strategies for obstetrical disorders and their complications [5,8,23]. In our previously published study, we have shown that four machine learning-based algorithms, which included clinical and paraclinical data recorded in the first trimester of pregnancy, had good overall predictive performance for the prediction of preeclampsia [23].

This study aimed to determine and compare the predictive performance of four machine learning-based algorithms for the prediction of preeclampsia, IUGR, and their association in a cohort of singleton pregnancies with at least one risk factor for ischemic placental disease.

## 2. Materials and Methods

This prospective study was conducted at “Cuza Voda” Clinical Hospital of Obstetrics and Gynecology, Iasi, Romania, between 3 January 2023 and 10 September 2023. The study included pregnant patients with singleton pregnancies and at least 1 risk factor for ischemic placental disease from the following list: maternal age >35 years old, smoking habit, obesity (body mass index, BMI > 25 kg/m^2^), family or personal history of preeclampsia or IUGR, maternal comorbidities (diabetes mellitus, chronic kidney disease, chronic hypertension, and autoimmune disorders), and who underwent a conventional first trimester down syndrome screening between 11 and 13 + 6 weeks of gestation.

The exclusion criteria comprised multifetal gestations, maternal age under 18 years old, incorrect first-trimester dating of the gestational age, first and second-trimester abortions, fetal death in utero, loss of follow-up, incomplete medical records, or the mother’s inability to offer informed consent.

The study was conducted during the project “Net4SCIENCE: Network for Applied Doctoral and Postdoctoral Research in the Fields of Smart Specialization—Health and Bioeconomy”, project code SMIS: 154722, and ethical approval for this study was obtained from the Institutional Ethics Committee of the University of Medicine and Pharmacy ‘Grigore T. Popa’ (No. 151/13 February 2022).

All patients underwent ultrasound examination, between 11 + 0 and 13 + 6 weeks of gestation, by certified obstetricians using an E8/E10 (General Electric Healthcare, Zipf, Austria) scanner for the measurement of crown–rump length (CRL), uterine artery pulsatility index (UtA-PI), and nuchal translucency (NT).

We collected a blood sample of 5 mL from all participants included in the study, which was stored at −20 °C until processing. From the extracted serum, we determined values of the following markers: β-HCG, PAPP-A, PlGF (using Brahms Kriptor analyzer, Thermo Fisher Scientific, Karlsruhe, Germany), and PP-13 (quantitative sandwich enzyme-linked immunosorbent assay—ELISA). The serum values of these markers were transformed into multiple of medians (MoM).

Using a calibrated device (Omron M3 COMFORT; Omron Corp, Kyoto, Japan), blood pressure was measured in accordance with the Fetal Medicine Foundation (FMF) recommendations, and the mean arterial pressure (MAP) was noted and transformed into MoMs [24].

In total, 245 patients were monitored during pregnancy, but only data from 210 patients was analyzed in this study due to a lack of information about the pregnancy outcomes. The following data was recorded from all patients: demographic data, BMI, smoking status, personal and family history of adverse pregnancy outcomes (PE, IUGR, preterm birth, abruptio placentae, etc.), maternal comorbidities, gestational age at the onset of PE/IUGR, gestational age at birth, birthweight, Apgar score, and adverse neonatal outcomes such as neonatal intensive care unit admission (NICU), intraventricular hemorrhage, necrotizing enterocolitis, or the need of invasive ventilation.

Preeclampsia was defined according to the new ISSHP recommendations [1] as gestational hypertension accompanied by one or more of the following new-onset conditions at ≥20 weeks’ gestation: (a) proteinuria; (b) other maternal end-organ dysfunction (neurological complications, pulmonary edema, hematological complications, acute kidney injury, or liver impairment); (c) uteroplacental dysfunction.

IUGR was defined and classified using the Delphi consensus [25], which is based on EFW and abnormal fetoplacental under the following Doppler parameters: early IUGR (<32 weeks of gestation) and late IUGR (≥32 weeks of gestation).

The following groups were examined, which corresponded to the main outcomes of our study: preeclampsia group (n = 11 patients), IUGR group (n = 15 patients), PE + IUGR (n = 4 patients), and control group (n = 180 patients). Additionally, we evaluated the predictive performance of machine learning algorithms for early (n = 6 patients) and late IUGR (n = 9 patients).

In the first phase of our analysis, we used descriptive statistics and a comparison of categorical variables (Pearson’s χ^2^ test) or continuous variables (ANOVA with the Bonferroni post hoc test) between our groups. A *p*-value less than 0.05 was considered statistically significant. These analyses were performed using STATA SE (version 17, 2023, StataCorp LLC, College Station, TX, USA).

In the second phase of our analysis, we constructed 4 predictive models based on machine learning, decision tree (DT), naïve Bayes (NB), support vector machine (SVM), and random forest (RF), which included the following data: maternal characteristics (age, BMI, nulliparity, type of conception, smoking status (yes/no)), personal or family history of adverse pregnancy outcomes (preeclampsia, intrauterine growth restriction, abruptio placentae, preterm birth, autoimmune disorders, chronic hypertension (yes/no)) comorbidities (chronic kidney disease, diabetes, chronic hypertension, and cardiovascular disorders), MAP, and values of serum biomarkers such as β-HCG, PAPP-A, PlGF, and PP-13. The data were segregated into 70% testing and 30% training and underwent 5-fold cross-validation.

A sensitivity analysis was performed to characterize the predictive performance of these models for PE, IUGR, PE + IUGR, early IUGR, and late IUGR. The models were constructed and analyzed using Matlab (version R2023a, The MathWorks, Inc., Natick, MA, USA).

## 3. Results

In the first step of our analysis, we comparatively evaluated the demographic and clinical characteristics of 210 pregnant patients (Table 1). Our results indicated that smoking during pregnancy was significantly more frequently encountered in the group of patients who later developed IUGR (n = 6, 40%, *p* = 0.004) in comparison with other groups. The patients in this group had also a significant personal history of autoimmune disorders (n = 3, 20%*, p* < 0.001) and adverse pregnancy outcomes (n = 3, 20%, *p* = 0.007) compared to other groups.

On the other hand, patients who later developed PE presented a significant personal history of chronic hypertension (n = 4, 36.3%, *p* < 0.001) and chronic kidney disease (n = 1, 9%, *p* = 0.001). Moreover, patients who were later diagnosed with PE and IUGR had a significantly higher prevalence of diabetes mellitus compared with other groups (n = 1, 25%, *p* = 0.01).

The examined groups were similar when we evaluated maternal age, mode of conception, parity, and BMI. Thus, we could not outline a significant statistical difference regarding these characteristics between groups.

Regarding the pharmacological interventions used, all patients with PE (11 patients) or PE associated with IUGR (4 patients) benefited from treatment with methyldopa (250 mg p.o.), and the dose was adjusted depending on the arterial pressure values. In cases with uncontrolled hypertension by methyldopa, we associated nifedipine with a delayed release (20 mg p.o.), and, if necessary, magnesium sulfate as guided by our local protocol.

**Figure 1:**
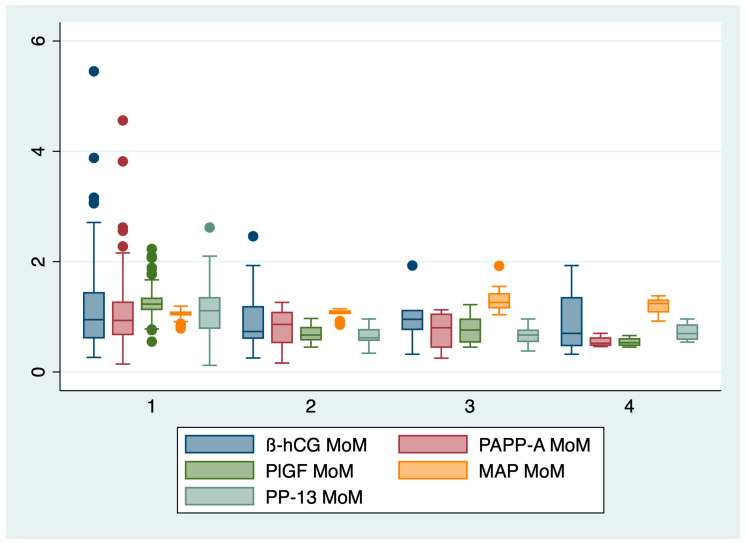
Boxplot representing the comparison of the first trimester markers between groups. 1—control group, 2—IUGR group, 3—PE group, 4—PE and IUGR group.

In the second step of our analysis, we compared the biochemical markers and mean arterial pressure between groups, and the results are presented in Table 2 and Figure 1.

The ANOVA analysis, followed by the Bonferroni post hoc test, indicated a statistically significant difference between the evaluated groups regarding the following markers: PAPP-A (*p* = 0.002), PlGF (*p* = 0.02), PP-13 *(p* < 0.001), and MAP *(p* < 0.001). The serum β-HCG levels determined in the first trimester of pregnancy did not significantly differ among the groups (*p* = 0.16).

The mean arterial pressure was significantly higher for patients who later developed preeclampsia (1.34 ± 0.24 MoM), while the serum values of PAPP-A, PlGF, and PP-13 were significantly lower for patients who later developed preeclampsia and/or IUGR in comparison with the control group.

In the third step of our analysis, we included the clinical characteristics and marker values determined in the first trimester of pregnancy into a database that was used for testing and training four machine learning-based algorithms. The results are expressed in Table 3.

When we evaluated the predictive performance of the four machine learning-based algorithms for the prediction of preeclampsia, we found that the RF algorithm obtained the best results, with a sensitivity (Se) of 90.9% (specificity (Sp) of 96.6%), false positive rate (FPR) of 3%, and accuracy of 96.3%. Both NB and SVM obtained similar results in terms of sensitivity (81.8%), specificity (95.5 versus 96.6%), and accuracy (94.7 versus 95.8%).

RF (Se—93.3%, Sp—96.1%, and accuracy—95.9%) and SVM (Se—93.3%, Sp—95.5%, and accuracy—95.3%) had similar performances for the prediction of IUGR, followed by NB (Se—86.6%, Sp—96.1%, and accuracy—95.3%) and DT (Se—73.3%, Sp—95%, and accuracy—93.3%).

Early onset IUGR was similarly predicted by all algorithms, but RF obtained the best results in terms of accuracy (96.2%). Late-onset IUGR was also best predicted by RF, with a Se of 88.8%, specificity of 95.5%, and accuracy of 95.2%.

Finally, all algorithms had a modest performance for the prediction of PE-IUGR association, but the best overall accuracies were achieved by NB and RF (95.1%).

Finally, we evaluated the main pregnancy outcomes among the groups, and the results are presented in Table 4. Neonates who were born from mothers diagnosed with preeclampsia or presented a growth restriction pattern during pregnancy had significantly more frequently adverse neonatal outcomes such as preterm birth (mostly iatrogenic), low Apgar score (seven or less), ARDS, invasive ventilation, and NICU admission (*p* < 0.001).

## 4. Discussion

The prediction of preeclampsia and IUGR remains a persistent challenge for obstetricians since these disorders are associated with important morbidity and mortality rates for both mothers and newborns. The classical screening program for these disorders, based only on maternal risk factors, has proven to be limited in terms of predictive performance, but it is still used in countries with limited financial resources [26]. Recent advances in screening strategies for these obstetrical disorders have increased the predictive performance and include a variety of markers [27]. The problem with this approach is the limited number of parameters included, as well as the high amount of human and financial resources required for their completion.

Every screening program can be improved, and in this study, we aimed to test the predictive performance of four machine learning-based algorithms for the prediction of PE and IUGR, as well as their association, which encompassed the maternal clinical characteristics, the serum biomarkers, and the MAP determined in the first trimester of pregnancy. We hypothesized that this approach would offer at least comparable results in terms of predictive performance as the combined screening strategies.

The RF algorithm showed superior performance for the prediction of all evaluated pathological categories including PE (accuracy: 96.3%), IUGR (accuracy: 95.9%), and its subtypes (early onset IUGR, accuracy: 96.2%, and late-onset IUGR, accuracy: 95.2%), as well as the association between PE and IUGR (accuracy: 95.1%). Both SVM and NB achieved a comparable performance in predicting IUGR (accuracy: 95.3%). However, SVM (accuracy: 95.8%) outperformed NB (accuracy: 94.7%) in predicting PE. The lowest predictive performance for all evaluated pathological categories was achieved by DT.

These results could be explained by the fact that RF is a complex algorithm, capable of operating with complex datasets, which demonstrated good overall predictive performance when used to predict obstetrical syndromes. For example, in a recent study by Melinte-Popescu et al., the authors indicated that RF achieved an accuracy of 92.8% for the prediction of PE [23]. Also, Liu et al. demonstrated in a retrospective study that RF outperformed other machine learning-based algorithms, such as DT and SVM, for the prediction of PE, with an accuracy of 74% [28].

Rescinito et al. conducted a systematic review and meta-analysis, analyzing data from 20 studies that examined the use of artificial intelligence/machine learning models for predicting IUGR [29]. The results of this analysis indicated that these techniques demonstrated a favorable overall diagnostic performance. Specifically, the sensitivity was found to be 0.84 (95% CI: 0.80–0.88), the specificity was 0.87 (95% CI: 0.83–0.90), the positive predictive value was 0.78 (95% CI: 0.68–0.86), and the negative predictive value was 0.91 (95% CI: 0.86–0.94). Furthermore, the researchers demonstrated that the combination of random forest and support vector machine (RF-SVM) yielded the highest level of accuracy (97%) in predicting intrauterine growth restriction [29].

Previous research has indicated that the prediction of early fetal growth restriction may pose greater challenges [30,31]. This phenomenon could be attributed to the possibility that certain risk factors linked to intrauterine growth restriction, such as maternal health conditions or placental abnormalities, may not manifest until the later stages of pregnancy. The machine learning algorithms we employed yielded comparable outcomes to traditional screening approaches for both early and late intrauterine growth restriction (IUGR) [31,32].

For individualized management, it is important to identify maternal risk factors for IUGR, PE, and other pregnancy complications [5,23]. In our study, we found out that the IUGR group had significantly more frequently a positive personal history of adverse pregnancy outcomes, autoimmune disease, and smoking habits in comparison to the other groups. On the other hand, patients who later developed PE presented a significant personal history of chronic hypertension and chronic kidney disease. These results are supported by recent literature data that outlined the impact of comorbidities and lifestyle on the occurrence of pregnancy complications.

Numerous biochemical and ultrasound markers have been proposed for the prediction of IUGR and PE, but only a few have achieved good predictive performance [20,33]. Our results indicated significantly lower serum values of PAPP-A, PlGF, and PP-13 for patients who later developed preeclampsia and/or IUGR in comparison with the control group, and are in line with the current literature data [34,35]. The mean arterial pressure was significantly higher for patients who later developed preeclampsia. On the other hand, the mean values of MAP and UtA-PI determined in the first trimester of pregnancy did not significantly differ between groups. This result could be explained by the small cohort of patients with heterogeneous characteristics.

Machine learning-based algorithms or other artificial intelligence-based methods could be used for the prediction of various disorders or complications, for the classification or diagnosis and surveillance, and can include a variety of parameters, from clinical data to biochemical and ultrasound markers, and proteomic and genomic data [36].

For example, a recent study by Gi et al. investigated the predictive performance of a machine learning model that was based on maternal risk factors, MAP, UtA-PI, PlGF, and pregnancy-associated plasma protein-A (PAPP-A) determined in the first trimester of pregnancy for the prediction of preeclampsia [37]. Their results indicated a similar performance of this model to the Fetal Medicine Foundation (FMF) model, with AUC values corresponding to early, preterm, and all types of preeclampsia of 0.920, 0.913, and 0.846.

In this prospective study, we chose to use an additional serum biomarker, PP-13. It is a member of the galectin family involved in spiral artery remodeling and placental inflammation and has demonstrated good predictive performance for IUGR and preeclampsia [20,21,22]. This biomarker is especially important for its high specificity as demonstrated in previous meta-analyses, and it can be used in various combined screening strategies for improving the overall accuracy of preeclampsia and IUGR prediction as early as the first trimester of pregnancy [20,38].

Asiltas and colleagues investigated the individual and combined predictive performance of PAPP-A, PP-13, β-HCG, and oxidative stress marker malondialdehyde (MDA) for the first-trimester prediction of PE [39]. Their results indicated that the serum’s PP-13 levels were significantly lower, while the serum’s MDA levels were significantly higher in cases who later developed PE. Moreover, these two biomarkers outperformed PAPP-A and β-HCG when used individually for the prediction of PE. A combination of MDA, PP-13, PAPP-A, and β-HCG achieved the highest predictive performance, with an AUC of 0.91, sensitivity of 97%, and sensitivity of 75%.

As far as we know, this would be the first prospective study that evaluated this particular combined screening approach on a cohort of singleton pregnancies. Nevertheless, it is important to interpret the findings of this study while taking into account certain limitations. These limitations include the relatively small sample size of patients, the inclusion of only a limited number of clinical and paraclinical characteristics, and the presence of imbalanced datasets. Machine learning algorithms possess the capability to effectively function with limited datasets, rendering them potentially valuable for risk stratification purposes. Conversely, the incorporation of predictive models grounded in machine learning techniques and the prospective design of our research are notable strengths.

Further studies on larger cohorts of singleton pregnancies could include various approaches of screening into machine learning-based algorithms and could offer a broader perspective on their predictive performance and utility for clinical practice.

## 5. Conclusions

Ischemic placental disease prediction is a vast field of research and innovative approaches are needed to optimize our current screening strategies.

PP-13 could be a potentially valuable serum biomarker for the first-trimester screening of preeclampsia and could aid in the risk stratification process of pregnant patients at risk.

Our approach included a combined screening strategy into four machine learning-based algorithms for the prediction of PE, IUGR, and their association, with the best predictive performance of these obstetrical disorders being achieved by RF.

Further studies could validate this approach on larger cohorts of patients, and our research could be used as a basis for an integrative perspective on ischemic placental disease prediction.

## Figures and Tables

**Table 1:** Clinical characteristics of the studied groups.

Clinical Characteristics	PE Group (n = 11 Patients)	IUGR Group (n = 15 Patients)	PE and IUGR Group (n = 4 Patients)	Control Group (n = 180 Patients)	*p*-Value
Age, years (mean ± SD)	29.6 ± 6.19	26.12 ± 5.74	27.32 ± 5.14	29.22 ± 5.31	0.52
Spontaneous conception (n/%)	Yes = 8 (72.7%)	Yes = 12 (80%)	Yes = 3 (75%)	Yes = 155 (86.1%)	0.56
IVF conception (n/%)	Yes = 1 (9%)	Yes = 2 (13.3%)	Yes = 1 (25%)	Yes = 15 (8.3%)	0.64
ICSI conception (n/%)	Yes = 2 (18.1%)	Yes = 1 (6.6%)	Yes = 0 (0%)	Yes = 10 (5.5%)	0.18
Nuliparous (n/%)	Yes = 6 (54.5%)	Yes = 10 (66.6%)	Yes = 2 (50%)	Yes = 95 (52.7%)	0.77
BMI, kg/m^2^, (mean and standard deviation)	25.86 ± 4.97	24.15 ± 5.01	23.62 ± 3.11	23.86 ± 4.16	0.15
Smoking habit (n/%)	Yes = 2 (18.1%)	Yes = 6 (40%)	Yes = 1 (25%)	Yes = 17 (9.4%)	0.004
Diabetes (n/%)	Yes = 1 (9%)	Yes = 1 (6.6%)	Yes = 1 (25%)	Yes = 3 (1.6%)	0.01
History of chronic hypertension (n/%)	Yes = 4 (36.3%)	Yes = 2 (13.3%)	Yes = 2 (50%)	Yes = 5 (2.7%)	<0.001
History of autoimmune disorders (n/%)	Yes = 1 (9%)	Yes = 3 (20%)	Yes = 1 (25%)	Yes = 3 (1.6%)	<0.001
Chronic kidney disease (n/%)	Yes = 1 (9%)	Yes = 0 (0%)	Yes = 0 (0%)	Yes = 4 (2.2%)	0.001
History of adverse pregnancy outcomes (n/%)	Yes = 2 (18.1%)	Yes = 3 (20%)	Yes = 1 (25%)	Yes = 11 (6.1%)	0.07

Legend: PE—preeclampsia, IUGR—intrauterine growth restriction, SD—standard deviation, n—number of patients, IVF—in vitro fertilization, ICSI—intracytoplasmic sperm injection, BMI—body mass index.

**Table 2:** Comparisons of the first-trimester biochemical markers and mean arterial pressure between groups.

Marker	PE Group (n = 11 Patients)	IUGR Group (n = 15 Patients)	PE and IUGR Group (n = 4 Patients)	Control Group (n = 180 Patients)	*p*-Value
β-HCG, MoM (mean ± SD)	0.96 ± 0.41	0.96 ± 0.65	0.91 ± 0.70	1.14 ± 0.75	0.16
PAPP-A, MoM (mean ± SD)	0.73 ± 0.34	0.81 ± 0.34	0.55 ± 0.11	1.05 ± 0.57	0.002
PlGF, MoM (mean ± SD)	0.77 ± 0.27	0.69 ± 0.14	0.54 ± 0.09	1.27 ± 0.24	0.02
PP-13, MoM (mean ± SD)	0.67 ± 0.19	0.66 ± 0.17	0.72 ± 0.18	1.09 ± 0.44	<0.001
MAP, MoM (mean ± SD)	1.34 ± 0.24	1.06 ± 0.09	1.20 ± 0.19	1.06 ± 0.06	<0.001

Legend: PE—preeclampsia, IUGR—intrauterine growth restriction, SD—standard deviation, n—number of patients, MoM—multiples of the median, β-HCG—beta-human chorionic gonadotropin, PAPP-A—pregnancy-associated plasma protein-A, PlGF—placental growth factor, PP-13—placental protein 13, MAP—mean arterial pressure.

**Table 3:** Predictive performance of four machine learning-based algorithms for the prediction of PE, IUGR, and their association.

ML Model	Groups	Se (%)	Sp (%)	FPR (%)	Matthews Coefficient	Accuracy (%)	Precision	F1 Score
DT	IUGR (15 patients)	73.3	95	5	0.45	93.3	0.55	0.62
	Early IUGR (6 patients)	83.3	93.8	6	0.48	93.5	0.31	0.45
	Late IUGR (9 patients)	77.7	93.3	6	0.50	92.6	0.36	0.50
	PE (11 patients)	72.7	94.4	5	0.55	94	0.44	0.55
	PE + IUGR (4 patients)	75	94.4	5	0.76	94	0.23	0.35
NB	IUGR (17 patients)	86.6	96.1	3	0.72	95.3	0.65	0.74
	Early IUGR (6 patients)	83.3	96.1	3	0.57	95.7	0.41	0.55
	Late IUGR (11 patients)	77.7	94.4	5	0.53	93.6	0.41	0.53
	PE (11 patients)	81.8	95.5	4	0.63	94.7	0.52	0.64
	PE + IUGR (4 patients)	75.1	95.5	4	0.43	95.1	0.27	0.40
SVM	IUGR (17 patients)	93.3	95.5	4	0.74	95.3	0.63	0.75
	Early IUGR (6 patients)	83.3	95	5	0.52	94.6	0.35	0.50
	Late IUGR (11 patients)	66.6	94.4	5	0.46	93.1	0.37	0.48
	PE (11 patients)	81.8	96.6	3	0.67	95.8	0.6	0.69
	PE + IUGR (4 patients)	0.75	0.95	5	0.41	94.5	0.25	0.37
RF	IUGR (17 patients)	93.3	96.1	3	0.76	95.9	0.66	0.77
	Early IUGR (6 patients)	83.3	96.7	3	0.59	96.2	0.83	0.58
	Late IUGR (11 patients)	88.8	95.5	4	0.64	95.2	0.5	0.64
	PE (11 patients)	90.9	96.6	3	0.73	96.3	0.62	0.74
	PE + IUGR (4 patients)	75	95.5	4	0.43	95.1	0.27	0.40

Table legend: IUGR—intrauterine growth restriction, PE—preeclampsia, DT—decision trees, NB—naïve Bayes, SVM—support vector machine, RF—random forest, Se—sensibility; Sp—specificity, FPR—False positive rate.

**Table 4:** Neonatal outcomes of the evaluated groups.

Neonatal Outcome	PE Group (n = 11 Patients)	IUGR Group (n = 15 Patients)	PE and IUGR Group (n = 4 Patients)	Control Group (n = 180 Patients)	*p*-Value
Preterm birth (n/%)	Yes = 8 (72.7%)	Yes = 12 (80%)	Yes = 3 (75%)	Yes = 25 (13.8%)	<0.001
Low-Apgar score (n/%)	Yes = 4 (36.3%)	Yes = 5 (33.3%)	Yes = 2 (50%)	Yes = 10 (5.5%)	<0.001
ARDS (n/%)	Yes = 3 (27.2%)	Yes = 4 (26.6%)	Yes = 2 (50%)	Yes = 8 (4.4%)	<0.001
Invasive ventilation (n/%)	Yes = 2 (18.1%)	Yes = 1 (6.6%)	Yes = 2 (50%)	Yes = 5 (2.7%)	<0.001
NICU admission (n/%)	Yes = 2 (72.7%)	Yes = 1 (80%)	Yes = 2 (75%)	Yes = 5 (13.8%)	<0.001

Legend: PE—preeclampsia, IUGR—intrauterine growth restriction, n—number of patients, ARDS—acute respiratory distress syndrome, NICU—neonatal intensive care unit admission.

## Data Availability

The datasets are part of Ingrid-Andrada Vasilache doctoral research and are available from this author upon a reasonable request.
